# Efficacy of Harmonic Scalpel Versus Bipolar Diathermy in Hemorrhoidectomy: A Systematic Review and Meta-Analysis of Nine Randomized Controlled Trials

**DOI:** 10.7759/cureus.34734

**Published:** 2023-02-07

**Authors:** Ebraheem Albazee, Abdulaziz Alenezi, Maryam Alenezi, Reham Alabdulhadi, Rawan J Alhubail, Khaled Ahmad Al Sadder, Fatma AlDabbous, Abdulrahman N Almutairi, Saad N Almutairi, Abdullah N Almutairi, Mujahed S Alenezi

**Affiliations:** 1 Department of Internship, Kuwait Institute for Medical Specializations, Kuwait City, KWT; 2 Department of General Medicine, Al Sabah Hospital, Kuwait City, KWT; 3 Department of General Surgery, Ministry of Health, Kuwait City, KWT; 4 Department of Medicine, University of Jordan, Amman, JOR; 5 Department of General Surgery, Al Sabah Hospital, Kuwait City, KWT

**Keywords:** systematic review and meta-analysis, harmonic, bipolar, haemorrhoid, piles

## Abstract

Hemorrhoidectomy is one of the most common surgical interventions to remove the third and fourth degrees of prolapse hemorrhoid. We carried out this systematic review and meta-analysis of the randomized controlled trials (RCTs) to comprehensively evaluate the efficacy of harmonic scalpel (HS) versus bipolar diathermy (BD) methods in terms of decreasing intraoperative and postoperative morbidities among patients undergoing hemorrhoidectomy. Suitable citations were found utilizing digital medical sources, including the CENTRAL, Web of Science, PubMed, Scopus, and Google Scholar, from inception until December 2022. Only RCTs that matched the inclusion requirements were selected. We used the updated Cochrane risk of bias (ROB) tool (version 2) to assess the quality of the involved citations. The Review Manager (version 5.4 for Windows) was used to perform the pooled analysis. Data were pooled and reported as mean difference (MD) or risk ratio (RR) with a 95% confidence interval (CI) in random-effects models. Overall, there was no significant difference between HS and BD in terms of decreasing intraoperative morbidities like operative time, intraoperative blood loss, mean duration of hospital stay, and mean duration of first bowel movement (P>0.05). Similarly, the rate of postoperative complications like pain, bleeding, urinary retention, anal stenosis, flatus incontinence, and wound edema; was similar in both groups with no significant difference (P>0.05). In conclusion, our pooled analysis revealed there was no substantial difference between HS and BD in terms of intraoperative and postoperative endpoints. Additional RCTs with larger sample sizes are needed to consolidate the power and quality of the presented evidence.

## Introduction and background

Hemorrhoidectomy is one of the most common surgical interventions to remove the third and fourth degrees of prolapse hemorrhoid [1]. Despite being a minor surgery, intraoperative and postoperative morbidities like blood loss, operative time, pain, anal stenosis, urinary retention, hemorrhage, and incontinence are considered a main concern [2]. Therefore, several tools have been introduced to decrease these intraoperative and postoperative morbidities, like a harmonic scalpel (HS), bipolar diathermy (BD), laser, and LigaSure (Medtronic, Dublin, Ireland)[3].

Traditional hemorrhoidectomy involving both closed and open techniques is considered the gold standard for prolapse piles globally. Recent studies showed the superiority of other techniques like HS and BD over traditional surgery [4,5]. A bipolar diathermy device is an anti-hemorrhagic tool and can deliver a precise amount of electrocautery energy across vascular structures with minimal surrounding thermal spread [3]. On the other hand, the harmonic scalpel method utilizes an ultrasonic blade vibrating at 55 kHz to concurrently dissect and coagulate soft tissues [5]. Collectively, both methods aim to minimize thermal-related damage to soft tissues and improve precision cutting.

Several randomized controlled trials (RCTs) have scrutinized the impact of HS versus BD methods for hemorrhoidectomy [6-14]. Nonetheless, the conclusions have been indecisive, owing to limitations of small sample size and inconsistent results. Thus, we carried out this systematic review and meta-analysis of RCTs to comprehensively evaluate the efficacy and safety of HS versus BD methods in terms of decreasing intraoperative and postoperative morbidities among patients undergoing hemorrhoidectomy. 

## Review

Review methodology

This meta-analysis of RCTs followed strictly the rules and steps in the Preferred Reporting Items for Systematic Reviews and Meta-Analyses (PRISMA) statement [15] and the Cochrane Handbook for Systematic Reviews and Meta-analysis of Interventions [16]. This review was registered in the PROSPERO database [registration ID: CRD42023392697]. Our PICOS criteria comprised: (P): patients undergoing hemorrhoidectomy, (I): harmonic scalpel (HS), (C): bipolar diathermy (BD), (O): efficacy and safety endpoints, (S): randomized controlled trials (RCTs).

Data and study selection

From the inception till December 2022, we depended on searching through several databases for suitable RCTs, involving Cochrane, Web of Science (WOS), PubMed, Scopus, and Google Scholar. Our search strategy involved: (Hemorrhoidectomy OR Haemorrhoidectomy OR Hemorrhoid* OR “Hemorrhoid Surgery” OR “Piles Surgery” OR Piles) AND (Harmonic OR “Harmonic Scalpel” OR “Ultrasonic Scalpel” OR “Ultrasonic Harmonic Scalpel”) AND (Bipolar OR “Bipolar Diathermy” OR “Bipolar cautery” OR “Bipolar Electrocautery”). Table 1 shows the exact literature search for each database.

**Table 1:** The exact literature search strategy used in every database

[PubMed] Randomized clinical trials: (Hemorrhoidectomy OR Haemorrhoidectomy OR Hemorrhoid* OR “Hemorrhoid Surgery” OR “Piles Surgery” OR Piles) AND (Harmonic OR “Harmonic Scalpel” OR “Ultrasonic Scalpel” OR “Ultrasonic Harmonic Scalpel”) AND (Bipolar OR “Bipolar Diathermy” OR “Bipolar cautery” OR “Bipolar Electrocautery”).
[Scopus] Article title, Abstract, Keywords: (Hemorrhoidectomy OR Haemorrhoidectomy OR Hemorrhoid* OR “Hemorrhoid Surgery” OR “Piles Surgery” OR Piles) AND (Harmonic OR “Harmonic Scalpel” OR “Ultrasonic Scalpel” OR “Ultrasonic Harmonic Scalpel”) AND (Bipolar OR “Bipolar Diathermy” OR “Bipolar cautery” OR “Bipolar Electrocautery”).
[Web of Science] All Fields: (Hemorrhoidectomy OR Haemorrhoidectomy OR Hemorrhoid* OR “Hemorrhoid Surgery” OR “Piles Surgery” OR Piles) AND (Harmonic OR “Harmonic Scalpel” OR “Ultrasonic Scalpel” OR “Ultrasonic Harmonic Scalpel”) AND (Bipolar OR “Bipolar Diathermy” OR “Bipolar cautery” OR “Bipolar Electrocautery”).
[Cochrane CENTRAL] Title Abstract Keyword: (Hemorrhoidectomy OR Haemorrhoidectomy OR Hemorrhoid* OR “Hemorrhoid Surgery” OR “Piles Surgery” OR Piles) AND (Harmonic OR “Harmonic Scalpel” OR “Ultrasonic Scalpel” OR “Ultrasonic Harmonic Scalpel”) AND (Bipolar OR “Bipolar Diathermy” OR “Bipolar cautery” OR “Bipolar Electrocautery”).
[Google Scholar] All Fields: (Hemorrhoidectomy OR Haemorrhoidectomy OR Hemorrhoid* OR “Hemorrhoid Surgery” OR “Piles Surgery” OR Piles) AND (Harmonic OR “Harmonic Scalpel” OR “Ultrasonic Scalpel” OR “Ultrasonic Harmonic Scalpel”) AND (Bipolar OR “Bipolar Diathermy” OR “Bipolar cautery” OR “Bipolar Electrocautery”).

To broaden the literature search, we scanned the reference lists of eligible studies and contemporary reviews for potentially missed relevant studies. The study selection process comprised omitting duplicate citations, followed by a screening of titles/abstracts, and then concluded with the full-text reading of the potential citations. Two coauthors independently completed the search strategy and selected studies; discrepancies were settled by consultation with the principal investigator.

Risk of bias assessment and data extraction

To rate the quality of the citations that were included, we used the Cochrane Risk of Bias checklist (the updated version) [17]. Two co-authors completed the risk of bias assessment, and discrepancies were established by a discussion with the principal investigator. Each scale domain and the overall quality of the chosen publications were given a risk level from “low risk of bias”, with “some concerns risk of bias”, to “high risk of bias” by the authors. Conflicts were resolved through discussions.

The first three categories of data were collected. First, we made a summary list of the features and characteristics of the citations that were involved, such as the trial identification (ID), country, duration, the total number of the sample size, and RCTs arms. Second, we obtained data on the fundamental details of the patients undergoing the intervention or control groups, such as sample size, age (years), sex, pain assessment tool, and follow-up duration. Third, we collected data on effectiveness outcomes, including operative time (minutes, intraoperative blood loss (ml), mean duration of hospital stay (days), mean duration of first bowel movement (days), and postoperative pain (10-points scale). Also, we gathered information on the rate of postoperative complications like bleeding, urinary retention, anal stenosis, flatus incontinence, and wound edema.

Certainty of evidence

We used the Grading of Recommendations Assessment, Development, and Evaluation (GRADE) method to grade the overall certainty of the evidence for each outcome.

Data analysis

The Review Manager program (Windows as version 5.4 of RevMan) was used for the pooled analysis. We collected the binary and continuous outcomes under the random-effects model for calculating the risk ratio (RR) and mean difference (MD) with a 95% confidence interval (Cl), respectively. The analysis depended on Inverse-Variance and Mantel-Haenszel techniques for our analyses. Heterogeneity was assessed by using the chi-square tests. Substantial heterogeneity was observed when the chi-square test with p<0.1 and the I2 test >50 [18]. For all endpoints, statistical significance was determined as p<0.05. Subgroup analyses were performed on postoperative pain according to the postoperative days (day 1, day 2, and day 7).

Results

Results of Literature Search

**Figure 1:**
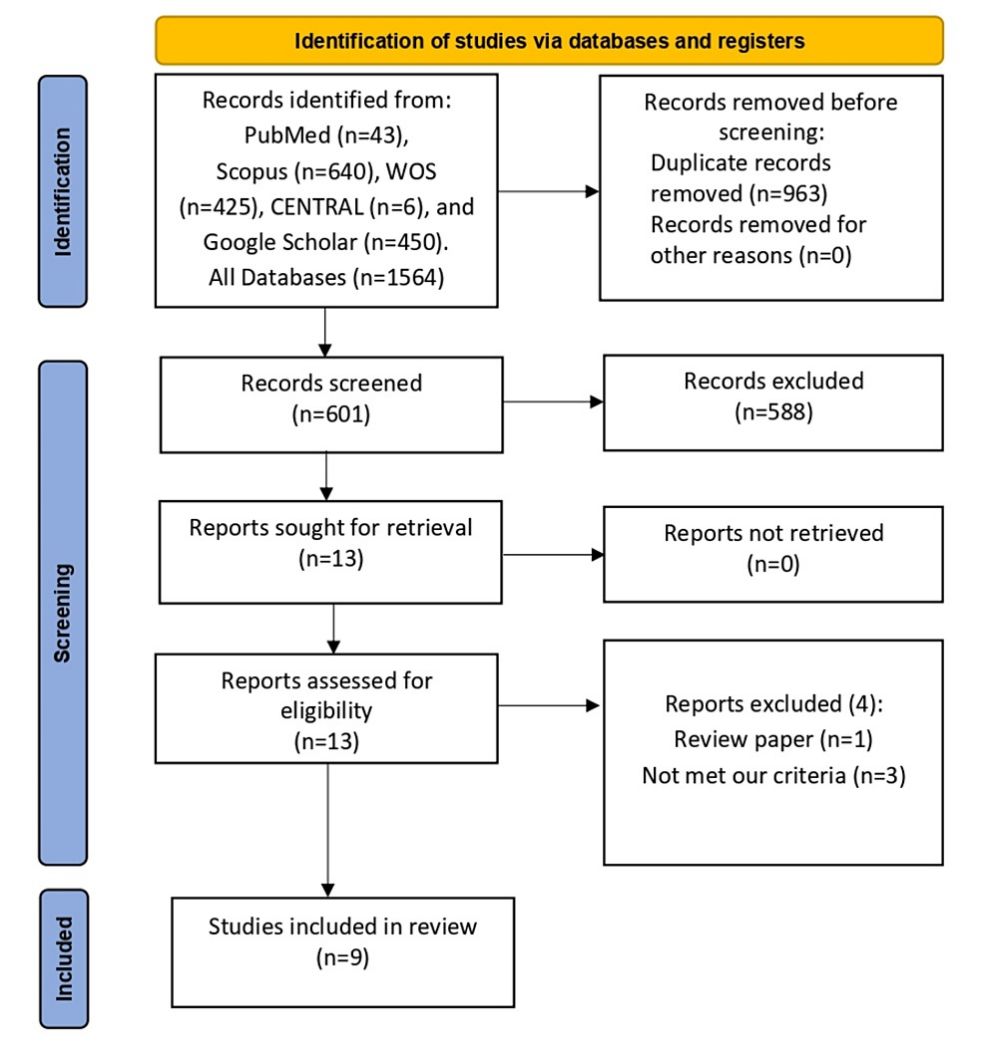
PRISMA flow diagram. [6-14]

Our search returned 601 articles after omitting 963 duplicated citations. Thereafter, during the title and abstract screening, also; 588 citations were excluded. Finally, following the exclusion of four citations during full-text screening, nine RCTs [6-14] met our PICOS requirements. The PRISMA diagram for our search procedure is shown in Figure 1, a total of 767 patients participated in these investigations, 328 were allocated to the HS group, and 385 were allocated to the BD group.

Study Characteristics

All the RCTs were executed in five countries, namely; Egypt, China, India, Pakistan, and Japan. Only patients with hemorrhoids grade III and IV were eligible for hemorrhoidectomy among the included trials. The follow-up duration ranged from 48 hours to one year. Eight out of nine RCTs [6-11,13,14] used a visual analogue scale (VAS) as a pain measurement tool, and only one trial [12] used a numeric rating scale (NRS) as a pain measurement tool; however, both tools were 10-points scale (0=no pain, and 10=severe pain). Tables 2, 3 depicts the summary and baseline characteristics of the included trials.

**Table 2:** Summary of the included trials. [6-14]

Study ID	Country	Duration	Total sample size, n	Trial arms		Grades of hemorrhoids
				Intervention	Control	
Abo-hashem et al. 2010 [6]	Egypt	July 2007-December 2008	n=64	Harmonic Scalpel	Bipolar diathermy	(III + IV)
Chung et al. 2002 [7]	China	April 1999-January 2001	n=59	Harmonic Scalpel	Bipolar diathermy	(III + IV)
Mashal et al. 2018 [8]	Egypt	July 2014-July 2016	n=90	Harmonic Scalpel	Bipolar diathermy	(III + IV)
Sarkar et al. 2018 [9]	India	September 2016-December 2017	n=60	Harmonic Scalpel	Bipolar diathermy	(III + IV)
Shaikh et al. 2021 [10]	Pakistan	January 2020-June 2020	n=128	Harmonic Scalpel	Bipolar diathermy	(III + IV)
Shoukat et al. 2016 [11]	Pakistan	April 2014-October 2014	n=130	Harmonic Scalpel	Bipolar diathermy	(III + IV)
Tsunoda et al. 2011 [12]	Japan	February 2010-December 2010	n=60	Harmonic Scalpel	Bipolar diathermy	(III + IV)
Ul Bari et al. 2023 [13]	India	July 2017-June 2019	n=64	Harmonic Scalpel	Bipolar diathermy	(III + IV)
Tahir Ullah et al. 2020 [14]	Pakistan	March 2016-April 2017	n=112	Harmonic Scalpel	Bipolar diathermy	(III + IV)

**Table 3:** Baseline characteristics of the included trials. [6-14]

Study ID	Group	Sample size, n	Age (years)	Sex, n	Pain assessment tool	Follow-up
				[male/female]		
Abo-hashem et al. 2010 [6]	Harmonic Scalpel	n=32	46 ±3.2	[20/12]	Visual analogue scale (10-points)	6 weeks
	Bipolar diathermy	n=32	44 ±2.1	[18/14]		
Chung et al. 2002 [7]	Harmonic Scalpel	n=29	49 ±14.9	[13/16]	Visual analogue scale (10-points)	12 weeks
	Bipolar diathermy	n=30	50.7 ±12.2	[16/14]		
Mashal et al. 2018 [8]	Harmonic Scalpel	n=45	31.2 ±5.4	[34/11]	Visual analogue scale (10-points)	4 weeks
	Bipolar diathermy	n=45	31.2 ±5.4	[39/6]		
Sarkar et al. 2018 [9]	Harmonic Scalpel	n=30	Not reported	Not reported	Visual analogue scale (10-points)	6 weeks
	Bipolar diathermy	n=30	Not reported	Not reported		
Shaikh et al. 2021 [10]	Harmonic Scalpel	n=64	41 ±7.2	[52/12]	Visual analogue scale (10-points)	6 weeks
	Bipolar diathermy	n=64	41 ±7.2	[45/19]		
Shoukat et al. 2016 [11]	Harmonic Scalpel	n=65	36 ±12.36	Not reported	Visual analogue scale (10-points)	1 week
	Bipolar diathermy	n=65	38 ±12.84	Not reported		
Tsunoda et al. 2011 [12]	Harmonic Scalpel	n=30	63 (3-85)	[16/14]	Numeric rating scale (10-points)	6 weeks
	Bipolar diathermy	n=30	63 (3-85)	[16/14]		
Ul Bari et al. 2023 [13]	Harmonic Scalpel	n=31	31.9 ±13.06	[20/11]	Visual analogue scale (10-points)	1 year
	Bipolar diathermy	n=33	32.4 ±14.34	[23/10]		
Tahir Ullah et al. 2020 [14]	Harmonic Scalpel	n=56	42 ±12.84	[22/34]	Visual analogue scale (10-points)	48 hours
	Bipolar diathermy	n=56	43 ±12.36	[24/32]		

Risk of Bias Assessment of Studies

**Figure 2:**
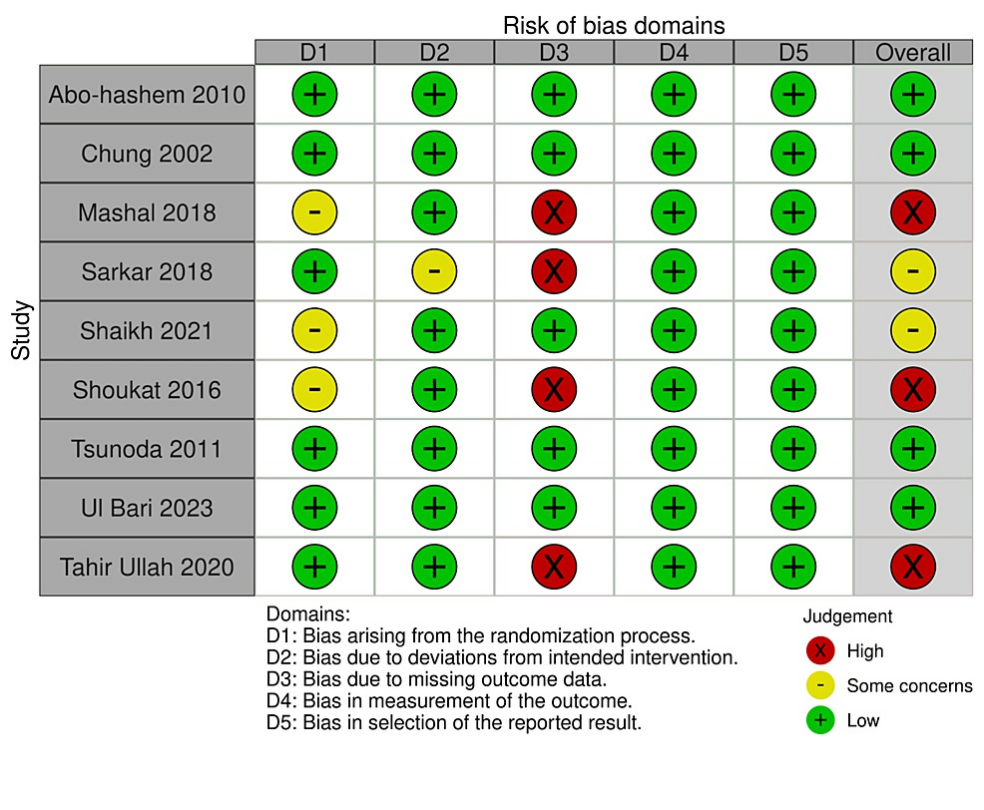
Risk of bias summary. [6-14]

**Figure 3:**
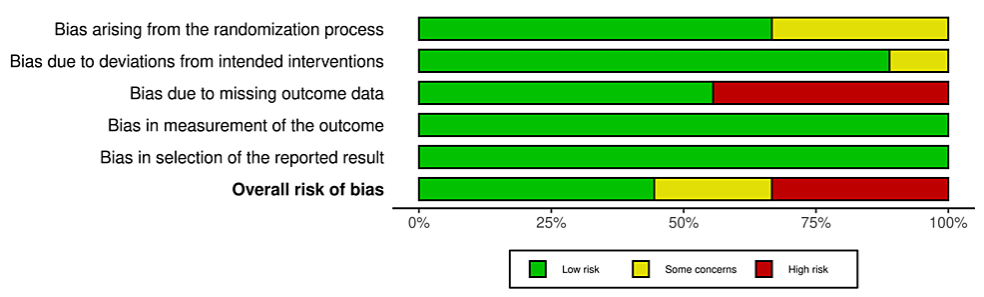
Risk of bias graph. [6-14]

Figures 2, 3 show the risk of bias assessment of the eligible RCTs-four RCTs [6,7,12,13] were assessed as having a “low” risk of bias. However, two RCTs [9,10] were considered as having “some concerns” risk of bias because Shaikah et al. [10] did not provide any data about the process of randomization, and Sarkar et al. [9] the surgeon was not blinded for the assigned groups. Furthermore, three RCTs [8,11,14] were evaluated as having a “high” risk of bias because they provide no information about some important outcomes, like the complication endpoints.

Results of the Meta-Analysis and Certainty of Evidence

**Figure 4:**
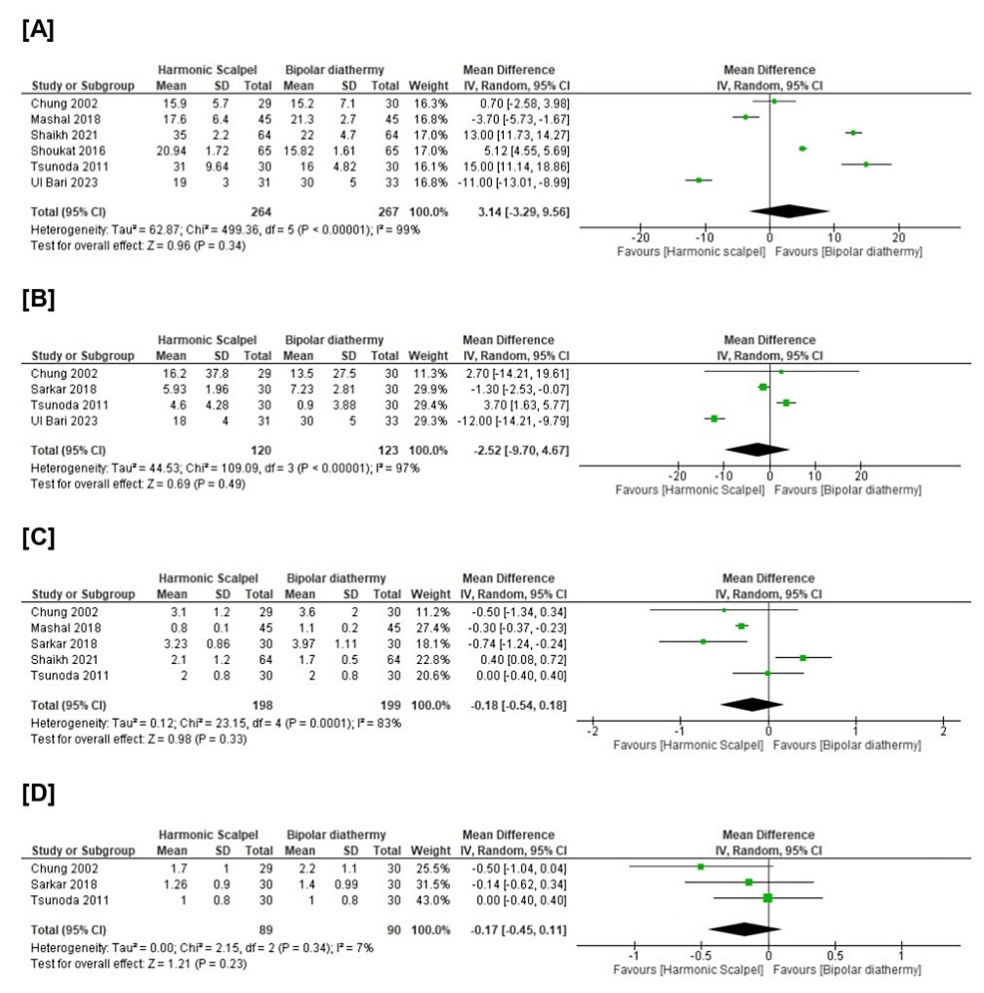
Meta-analysis of the mean change in [A] operative time (minutes), [B] intraoperative blood loss (ml), [C] duration of hospital stays (days), [D] duration of first bowel movement (days). [6-14]

Surprisingly, there was no significant difference between HS and BD groups in terms of operative time (n=6 RCTs, MD=3.14, 94% CI [-3.29, 9.56], p=0.34), intraoperative blood loss (n=4 RCTs, MD=-2.25, 95% CI [-9.70, 4.67], p=0.49), mean duration of hospital stay (n=5 RCTs, MD=-0.18, 95% CI [-0.54, 0.18], p=0.33), and mean duration of first bowel movement (n=3 RCTs, MD=-0.17, 95% CI [-0.45, 0.11], p=0.23). All pooled analyses were heterogeneous (chi-square p<0.1, I-square>50%). Figure 4 and Table 4.

**Figure 5:**
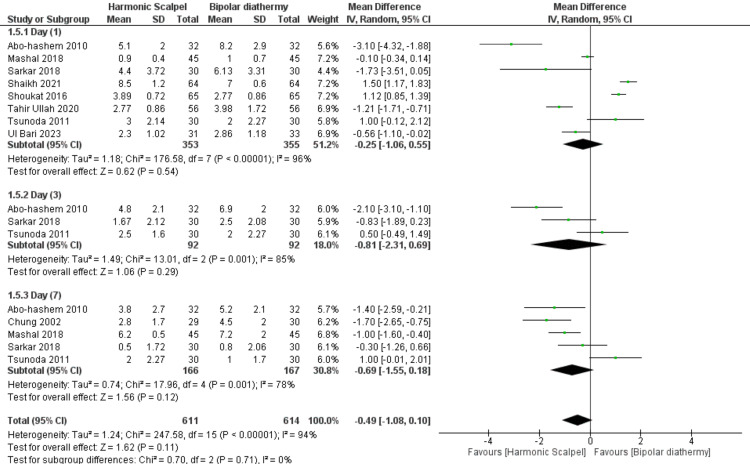
Meta-analysis of the mean change in postoperative pain (10-points scale). [6-14]

Furthermore, regarding postoperative pain overall assessment, there was no significant difference between HS and BD groups (n=9 RCTs, MD=-0.49, 95% CI [-1.08, 0.10], p=0.11). Also, there was no significant difference between HS and BD groups on day 1 (n=8 RCTs, MD=-0.25, 95% CI [-1.06, 0.55], p=0.54), day 3 (n=3 RCTs, MD=-0.81, 95% CI [-2.31, 0.69], p=0.29), and day 7 (n=5 RCTs, MD=-0.69, 95% CI [-1.55, 0.18], p=0.12). All pooled analyses were heterogeneous (chi-square p<0.1, I-square>50%). Figure 5 and Table 4. 

**Figure 6:**
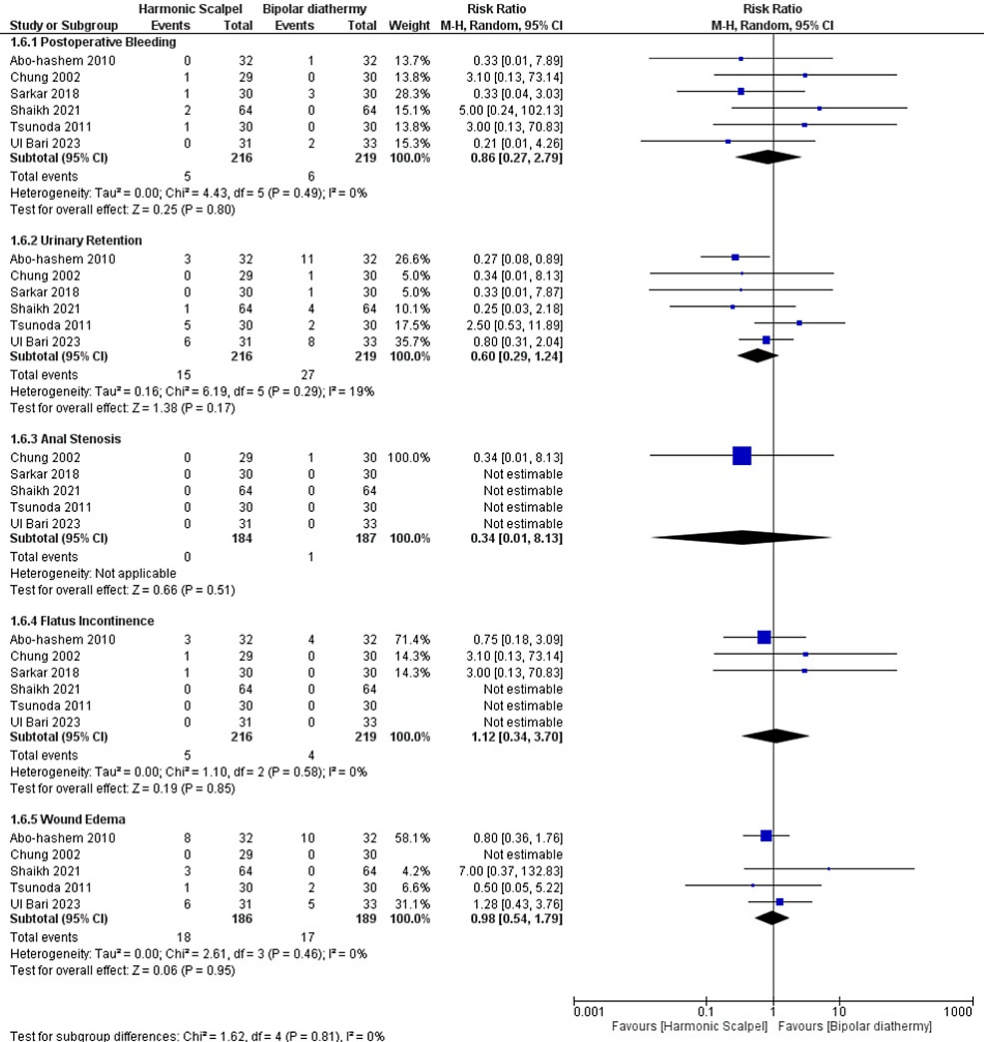
Meta-analysis of the rate of postoperative complications. [6-14]

Regarding the rate of postoperative complications, similarly and interestingly, there was no significant difference between HS and BD on the rate of postoperative complications, namely- bleeding (n=6 RCTs, RR=0.86, 95% CI [0.27, 2.79], p=0.80), urinary retention (n=6 RCTs, RR=0.60 95% CI [0.29, 1.24], p=0.17), anal stenosis (n=5 RCTs, RR=0.34, 95% CI [0.01, 8.13], p=0.51), flatus incontinence (n=6 RCTs, RR=1.12, 95% CI [0.34, 3.70], p=0.85), and wound edema (n=4 RCTs, RR=0.98, 95% CI [0.54, 1.79], p=0.95). All pooled analyses were homogenous (chi-square p>0.1, I-square<50%). Figure 6 and Table 4. 

 

**Table 4:** Summary of GRADE rating.

Outcome	Participants (studies)	Risk of bias	Inconsistency	Indirectness	Imprecision	Other considerationsᵃ	Overall certainty of evidence
Operative time (min)	531 (6 RCTs)	seriousᵇ	seriousᶜ	not serious	not serious	not serious	⨁⨁◯◯ Low
Intraoperative blood loss (ml)	243 (4 RCTs)	not serious	seriousᶜ	not serious	seriousᵈ	not serious	⨁⨁◯◯ Low
Duration of hospital stays (days)	397 (5 RCTs)	seriousᵇ	seriousᶜ	not serious	not serious	not serious	⨁⨁◯◯ Low
Duration of first bowel movement (days)	179 (3 RCTs)	seriousᵇ	not serious	not serious	seriousᵈ	not serious	⨁⨁◯◯ Low
Postoperative pain (10-points)	531 (6 RCTs)	seriousᵇ	seriousᶜ	not serious	not serious	not serious	⨁⨁◯◯ Low
Postoperative bleeding (%)	435 (6 RCTs)	seriousᵇ	not serious	not serious	not serious	not serious	⨁⨁⨁◯ Moderate
Urinary retention (%)	435 (6 RCTs)	seriousᵇ	not serious	not serious	not serious	not serious	⨁⨁⨁◯ Moderate
Anal stenosis (%)	371 (5 RCTs)	seriousᵇ	not serious	not serious	not serious	not serious	⨁⨁⨁◯ Moderate
Flatus incontinence (%)	435 (6 RCTs)	seriousᵇ	not serious	not serious	not serious	not serious	⨁⨁⨁◯ Moderate
Wound edema (%)	375 (5 RCTs)	seriousᵇ	not serious	not serious	not serious	not serious	⨁⨁⨁◯ Moderate
ᵃ Other considerations are publication bias, large effect, dose response, and plausible confounding factors. ᵇ As the included studies showed higher risk of bias especially with randomization process besides other bias. ᶜ As the outcome had significant heterogeneity. ᵈ As the analysis included small number of patients with wide confidence interval. Moderate quality— Further research is likely to have an important impact on our confidence in the estimate of effect and may change the estimate. Low quality— Further research is very likely to have an important impact on our confidence in the estimate of effect and is likely to change the estimate.							

Discussion

Summary of the Review Findings

The current systematic review and meta-analysis of RCTs evaluated the efficacy of HS in comparison with BD among patients with a third and fourth degree of prolapsed hemorrhoid and undergoing hemorrhoidectomy. Overall, there was no significant difference between HS and BD in terms of decreasing intraoperative morbidities like operative time (minutes), intraoperative blood loss (ml), mean duration of hospital stay (days), and mean duration of first bowel movement (days) (P>0.05). Furthermore, postoperative pain included subgroups on day 1, day 3, and day 7; our pooled analysis revealed there was no difference between both groups. Similarly, the rate of postoperative complications like bleeding, urinary retention, anal stenosis, flatus incontinence, and wound edema; was similar in both groups with no significant difference (P>0.05).

Interpretation and the Review Findings and Clinical Significance

There is a growing body of research directed toward finding the most optimal surgical method to reduce the commonly encountered hemorrhoidectomy-associated complications, which include postsurgical pain, urinary retention, anal stenosis, hemorrhage in addition to operative time, and intraoperative blood loss. Reduction of these complications has been proven to hasten patient recovery, decrease healthcare expenses, and enhance the quality of well-being. In the present meta-analysis, there was disagreement between the included RCTs regarding intraoperative endpoints like intraoperative blood loss and operative time. For operative time as an example, Shaikah et al. [10], Shoukat et al. [11], and Tsunoda et al. [12] found the effect size favors the BD technique over HS. On the other hand, Mashal et al. [8] and Ul Bari et al. [13] found the effect size favors HS over BD. Interestingly, our pooled analysis revealed insignificant differences between both groups. Inconsistency between these studies can be ascribed to the bias of some of the included studies leading to differences in conclusions, as well as methodological and statistical heterogeneity. Moreover, an additional explanation of the observed heterogeneity might be the difference in surgical skills across the included studies and the difference in the baseline characteristics of patients. 

Postoperative pain is the most popular complication of surgical hemorrhoidectomy, which is present in most patients [19] and is caused by multiple factors: thermal, mechanical, and chemical. In our meta-analysis, both techniques showed there was no superiority for each one in comparison with the other one (Figure 4.). Balciscueta et al. [19] performed a network meta-analysis of RCTs to examine different hemorrhoidectomy techniques in terms of pain, and they found the conventional open hemorrhoidectomy was the most painful on the first and seventh postoperative days; however, the pain was reduced after closed hemorrhoidectomy technique and when bipolar diathermy or harmonic scalpel was used.

The current pooled analysis found no substantial variance and homogeneity between HS and BD groups among the included RCTs in the postoperative endpoints like bleeding, urinary retention, anal stenosis, flatus incontinence, and wound edema. Simillis et al. [20] reported that HS and LigaSure (Medtronic, Dublin, Ireland) have fewer postoperative clinical complications. Aibuedefe et al. [19] observed that infrared photocoagulation and LigaSure had fewer postoperative clinical complications. Many studies that compare LigaSure with conventional hemorrhoidectomy see fewer postoperative clinical complications [21].

Chen et al. [22] performed a meta-analysis of RCTs to compare LigaSure hemorrhoidectomy versus stapled hemorrhoidectomy in terms of clinical outcomes like postoperative pain, incontinence, and recurrence and postoperative complications like bleeding, anal stenosis, and difficulty of defecation. They concluded that LigaSure vessel sealing systems is superior to stapled in term of reducing operative time and the recurrence rate. However, there is no difference between these techniques regarding the other outcomes like postoperative pain, bleeding, incontinence, and returned to normal activities. Also, Zhang and colleagues [23] conducted a systematic review and meta-analysis of trials that examine the difference between LigaSure hemorrhoidectomy and the procedure for prolapse and hemorrhoids (PPH) in postoperative pain, the rate of recurrence, returned to normal activities, and postoperative complications. They found as an overall judgment, there was no difference between LigaSure and PPH techniques, but the recurrence rate was less in the LigaSure group. All in all, a previous meta-analysis that investigated the efficacy and safety of different techniques like conventional, LigaSure, stapled, Milligan-Morgan, as well as a harmonic scalpel and bipolar diathermy; have the same conclusions and recommendations, and are consistent with our findings [3,19-23].

According to cost, the use of BD was associated with a cost saving 790$ compared with HS during a hemorrhoidectomy procedure. The list price of the disposable electrode of the bipolar diathermy system (LS3111 mode) is approximately $224, and that of the disposable handpiece of the ultrasonic scalpel system (CS14C mode) is approximately $790 and represents a direct addition to the cost of the procedure [12].

Strengths and Limitations

To our knowledge, this is the first meta-analysis of RCTs that thoroughly investigated the efficacy of the harmonic scalpel versus bipolar diathermy hemorrhoidectomy techniques. In addition, subgroup analyses were conducted to better understand whether or not a certain technique exhibited superiority in a particular area. Nevertheless, this study is not without its limitations. Firstly, only nine RCTs with a relatively small sample size were included. In addition, there was a substantial extent of heterogeneity across the included data. Moreover, there were some discrepancies in the reported methodologies used across the studies for the same measured endpoint, including a different period regarding the follow-up duration.

## Conclusions

The current systematic review and meta-analysis of RCTs evaluated the efficacy and safety of HS in comparison with BD among patients with a third and fourth degree of prolapsed hemorrhoid and undergoing hemorrhoidectomy. The findings revealed there was no substantial difference between HS and BD in terms of intraoperative endpoints like operative time and intraoperative blood loss and postoperative endpoints like duration of hospital stay, duration of first bowel movement, pain, bleeding, anal stenosis, urinary retention, flatus incontinence, and wound edema.

## References

[1] MacRae HM, McLeod RS (1995). Comparison of hemorrhoidal treatment modalities. A meta-analysis. Dis Colon Rectum.

[2] Sanchez C, Chinn BT (2011). Hemorrhoids. Clin Colon Rectal Surg.

[3] Aibuedefe B, Kling SM, Philp MM, Ross HM, Poggio JL (2021). An update on surgical treatment of hemorrhoidal disease: a systematic review and meta-analysis. Int J Colorectal Dis.

[4] Onur Gülseren M, Dinc T, Özer V, Yildiz B, Cete M, Coskun F (2015). Randomized controlled trial comparing the effects of vessel sealing device and Milligan Morgan technique on postoperative pain perception after hemorrhoidectomy. Dig Surg.

[5] Mushaya CD, Caleo PJ, Bartlett L, Buettner PG, Ho YH (2014). Harmonic scalpel compared with conventional excisional haemorrhoidectomy: a meta-analysis of randomized controlled trials. Tech Coloproctol.

[6] Abo-hashem AA, Sarhan A, Aly AM (2010). Harmonic Scalpel compared with bipolar electro-cautery hemorrhoidectomy: a randomized controlled trial. Int J Surg.

[7] Chung CC, Ha JP, Tai YP, Tsang WW, Li MK (2002). Double-blind, randomized trial comparing Harmonic Scalpel hemorrhoidectomy, bipolar scissors hemorrhoidectomy, and scissors excision: ligation technique. Dis Colon Rectum.

[8] Mashal A, Magdy A (2018). Outcome of harmonic scalpel hemorrhoidectomy in comparison with bipolar diathermy. Ka Al-Ain Jr Surg.

[9] Sarkar A, Choksi DB, Sutaria A, Sindhal M (2018). Harmonic scalpel versus bipolar diathermy in Milligan-Morgan haemorrhoidectomy: a randomized controlled study. Int Surg J.

[10] Shaikh RA, Memon AZ, Dal AN, Ahmed A, Mangi MH (2021). Comparison of outcome of bipolar electrocautery versus harmonic scalpel in the management of third and fourth degree hemorrhoids. Ann PIMS.

[11] Shoukat H, Iqbal M, Ullah S, Mirza A, Dar UF, Dar UF (2016). Comparison of hemorrhoidectomy using bipolar diathermy vs harmonic scalpel. Pakistan J Med Heal Sci.

[12] Tsunoda A, Sada H, Sugimoto T, Kano N, Kawana M, Sasaki T, Hashimoto H (2011). Randomized controlled trial of bipolar diathermy vs ultrasonic scalpel for closed hemorrhoidectomy. World J Gastrointest Surg.

[13] Ul Bari S, Malik A, Kangoo A (2021). Harmonic scalpel hemorrhoidectomy versus bipolar diathermy hemorrhoidectomy - A prospective evaluation. Indian J Colo-Rectal Surg.

[14] Ullah T, Khan M, Ali A, Khan I, Younas G, Hameed A (2020). Efficacy of harmonic scalpel versus bipolar electrocautry in hemorroidectomy. Khyb Jr Med Sci.

[15] Page MJ, McKenzie JE, Bossuyt PM (2021). The PRISMA 2020 statement: an updated guideline for reporting systematic reviews. Syst Rev.

[16] Cumpston M, Li T, Page MJ, Chandler J, Welch VA, Higgins JP, Thomas J (2019). Updated guidance for trusted systematic reviews: a new edition of the Cochrane Handbook for Systematic Reviews of Interventions. Cochrane Database Syst Rev.

[17] Sterne JA, Savović J, Page MJ (2019). RoB 2: a revised tool for assessing risk of bias in randomised trials. BMJ.

[18] Grant J, Hunter A (2006). Measuring inconsistency in knowledgebases. J Intell Inf Syst.

[19] Balciscueta Z, Balciscueta I, Uribe N (2021). Post-hemorrhoidectomy pain: can surgeons reduce it? A systematic review and network meta-analysis of randomized trials. Int J Colorectal Dis.

[20] Simillis C, Thoukididou SN, Slesser AA, Rasheed S, Tan E, Tekkis PP (2015). Systematic review and network meta-analysis comparing clinical outcomes and effectiveness of surgical treatments for haemorrhoids. Br J Surg.

[21] Nienhuijs S, de Hingh I (2009). Conventional versus LigaSure hemorrhoidectomy for patients with symptomatic Hemorrhoids. Cochrane Database Syst Rev.

[22] Xu L, Chen H, Lin G, Ge Q (2015). LigaSure versus Ferguson hemorrhoidectomy in the treatment of hemorrhoids: a meta-analysis of randomized control trials. Surg Laparosc Endosc Percutan Tech.

[23] Zhang L, Xie Y, Huang D, Ma X, Wang W, Xiao H, Zhong W (2022). LigaSure hemorrhoidectomy versus the procedure for prolapse and hemorrhoids: A meta-analysis of randomized controlled trials. Medicine (Baltimore).

